# Oral hygiene and oral health in older people with dementia: a comprehensive review with focus on oral soft tissues

**DOI:** 10.1007/s00784-017-2264-2

**Published:** 2017-11-15

**Authors:** Suzanne Delwel, Tarik T. Binnekade, Roberto S. G. M. Perez, Cees M. P. M. Hertogh, Erik J. A. Scherder, Frank Lobbezoo

**Affiliations:** 1 Faculty of Behavioral and Movement Sciences, Department of Clinical Neuropsychology, VU University, Amsterdam, The Netherlands; 2 Department of Oral Kinesiology, Academic Centre for Dentistry Amsterdam (ACTA), Faculty of Dentistry, University of Amsterdam and Vrije Universiteit Amsterdam, Gustav Mahler Laan 3004, 1081 LA Amsterdam, The Netherlands; 3 Department of Anesthesiology and Amsterdam Public Health Research Institute, VU University Medical Centre, Amsterdam, The Netherlands; 4 Faculty of Medicine, Department of Elderly Care Medicine, VU University Medical Centre, Amsterdam, The Netherlands

**Keywords:** Dementia, Older people, Elderly, Aged, Gerodontology, Oral health, Oral hygiene, Stomatognathic disease

## Abstract

**Background:**

The number of older people with dementia and a natural dentition is growing. Recently, a systematic review concerning the oral health of older people with dementia with the focus on diseases of oral hard tissues was published.

**Objective:**

To provide a comprehensive literature overview following a systematic approach of the level of oral hygiene and oral health status in older people with dementia with focus on oral soft tissues.

**Methods:**

A literature search was conducted in the databases PubMed, CINAHL, and the Cochrane Library. The following search terms were used: dementia and oral health or stomatognathic disease. A critical appraisal of the included studies was performed with the Newcastle-Ottawa scale (NOS) and Delphi list.

**Results:**

The searches yielded 549 unique articles, of which 36 were included for critical appraisal and data extraction. The included studies suggest that older people with dementia had high scores for gingival bleeding, periodontitis, plaque, and assistance for oral care. In addition, candidiasis, stomatitis, and reduced salivary flow were frequently present in older people with dementia.

**Conclusions:**

The studies included in the current systematic review suggest that older people with dementia have high levels of plaque and many oral health problems related to oral soft tissues, such as gingival bleeding, periodontal pockets, stomatitis, mucosal lesions, and reduced salivary flow.

**Scientific rationale for study:**

With the aging of the population, a higher prevalence of dementia and an increase in oral health problems can be expected. It is of interest to have an overview of the prevalence of oral problems in people with dementia.

**Principal findings:**

Older people with dementia have multiple oral health problems related to oral soft tissues, such as gingival bleeding, periodontal pockets, mucosal lesions, and reduced salivary flow.

**Practical implications:**

The oral health and hygiene of older people with dementia is not sufficient and could be improved with oral care education of formal and informal caregivers and regular professional dental care to people with dementia.

**Electronic supplementary material:**

The online version of this article (10.1007/s00784-017-2264-2) contains supplementary material, which is available to authorized users.

## Introduction

Aging of the world population has occurred at an unprecedented rate in the twentieth century and is forecasted to increase further [1]. Given the increase of general health problems with aging [2], and the presence of many interactions between general health and oral health [3, 4], an increase of oral health problems is to be expected.

Common oral health problems in older people are caries, periodontitis, reduced salivary flow, candida, and mucosal lesions [5, 6]. In developed countries, caries has a high prevalence in older adults with a mean number of decayed and filled coronal surfaces ranging from 22 to 35 and a mean number of decayed and filled root surfaces ranging from 2.2 to 5.3 [6–8]. In developing countries, these data are scarce [6]. Furthermore, periodontal disease is frequently present in older adults [9]. Specifically, mild periodontitis, with periodontal pockets of 4–5 mm, is present in 62–97% of the older persons [9]. More severe periodontitis, with pockets of 6 mm or more, is present in 20–48% of the older persons [9]. In addition, the prevalence of oral dryness increases with age, affecting approximately 30% of the older adults [10, 11]. Salivary flowrate decrease can result in difficulties with swallowing, eating, and communication [11]. Moreover, reduced salivary flow can cause halitosis, a higher prevalence of inflammation of the mucosa and parotid, candidiasis, dental caries in dentate persons, and frictional lesions in denture wearers [11]. The majority of oral diseases, including oral cancer, occur in older adults [12]. Among oral mucosal lesions, denture-related lesions, such as stomatitis, angular cheilitis, ulcers, and hyperplasia, are most common [13]. The above-mentioned oral health problems do not only affect oral health and functioning but may also cause orofacial pain or discomfort, and can have a negative impact on the quality of life [14, 15].

Compared to older people who are cognitively intact, older people who develop dementia are at increased risk of establishing oral health problems, as a result of decline in self-care and motor skills [16, 17]. Conversely, tooth loss and periodontitis may be risk factors for cognitive decline, although the exact presence and causality of the association between oral health problems and the development of dementia remains unclear [18, 19].

Several studies described oral disease as a risk factor for the development of dementia, but did not provide separate oral health data for the group of participants with dementia [20–27]. It is important to have an up-to-date overview of the oral health of older people with already present dementia, because the number of older people with natural dentition and possible risk factors for dementia is still increasing. Therefore, the aim of this study was to provide an up-to-date overview of studies about oral health in people with dementia. A previous review focused on dental hard tissues [28] and this review will aim at the oral soft tissues and oral hygiene of older people with dementia.

## Material and methods

For this review, the PRISMA statement [29] was followed and a protocol for the review process was developed in advance. The main question for the review was what is the prevalence of oral health problems in older people with dementia? The subquestion was how is the oral health of older people with dementia, compared with older people without dementia? The search terms were oral health, stomatognathic disease, and dementia. No separate oral health terms were used for the main search. The multidisciplinary team consisted of a professor in dentistry (FL), a professor in neuropsychology (ES), a professor in geriatric medicine and ethics (CH), a professor in palliative care and specialist in methodology (RP), a dentist with experience in gerodontology (SD), and a neuropsychologist with experience with older people with dementia (TB).

## Criteria

Studies that were included were (randomized) controlled trials and observational studies with and without control groups (cohort, case-control, cross-sectional). Studies that were not included were reviews and case reports. The inclusion criteria for this study were diagnosis of dementia, age 60 years or older, useable quantitative data concerning oral health in a group of participants with dementia, and stomatognathic disease.

## Search

The search strategy was developed in collaboration with the university library of the VU Medical Centre in Amsterdam. The search was performed in PubMed, CINAHL, and the Cochrane Library. The last updated search was performed on 12 January 2017. In PubMed, the following search query was used: ((((“Oral Health”[Mesh] OR “Oral Health” [tiab])) OR “Stomatognathic Diseases”[Mesh])) AND ((“Dementia”[Mesh] OR “Dementia”[tiab])). In CINAHL: (((MH “Oral Health” OR TI”Oral Health” OR AB”Oral Health”) OR (MH “Stomatognathic Diseases+” OR TI”Stomatognathic Diseases” OR AB”Stomatognathic Diseases”)) AND (MH “Dementia+” OR TI “Dementia” OR AB”Dementia”)). In the Cochrane Library: (“Oral Health” or “Stomatognathic Diseases”) AND “dementia”. No limits were applied to the search for language, year of publication, or methodology.

## Study selection

The titles, abstracts, and full texts in Dutch, English, and German were screened independently by two reviewers (SD and TB) according to the pre-established protocol and the inclusion and exclusion criteria mentioned above. Disagreements between reviewers were resolved by consensus. Articles in languages other than Dutch, English, or German were assessed by a native speaker with a background in dentistry, after instruction by the authors. If the diagnosis dementia or data related to oral health were unclear, the corresponding authors were contacted, up to a maximum of three times over a period of 4 months. If this did not lead to usable data, the article was excluded.

## Critical appraisal

The risk of bias within studies was critically appraised by two reviewers (SD and TB). For cross-sectional, case-control, and cohort studies, the Newcastle-Ottawa scale (NOS) was used [30] and for (randomized) controlled trials the Delphi list [31]. The diagnosis of dementia was considered adequate if the following criteria for dementia diagnosis were used: the Diagnostic and Statistical Manual of Mental Disorders (DSM-III and IV) [32], International Classification of Disease (ICD-9 and 10) [33], and the Alzheimer’s criteria by the National Institute of Neurological and Communicative Disorders and Stroke and the Alzheimer’s disease and Related Disorders Association (NINCDS-ADRDA) [34, 35].

For studies with cases (with dementia) and controls (without dementia), the groups were considered comparable if the group means for age and gender were not statistically different at the .05 level.

The oral health examination was considered adequate if a structured dental examination by a dentist took place. The person who performed the oral health examination was considered ‘blinded’ if (s)he did not know the cognitive status of the participants in advance. The follow-up of cohort studies was considered long enough if it was longer than 3 months and considered adequate if not more than 20% of the participants was lost to follow-up.

## Data extraction

The data extraction was performed by one reviewer (SD) and was checked by two other reviewers (TB and FL). The following data was extracted: (1) participant characteristics, including age and dementia diagnosis, (2) study design, e.g., cross-sectional, case-control, cohort study, or (randomized) controlled trial, and (3) baseline outcome measures, including periodontal health and treatment need, oral hygiene, assistance need with oral care, oral mucosal status, and salivary flow. The principal outcome measures were means and percentages. The methodological and clinical heterogeneity of the data was checked.

## Results

### Search results

**Fig. 1:**
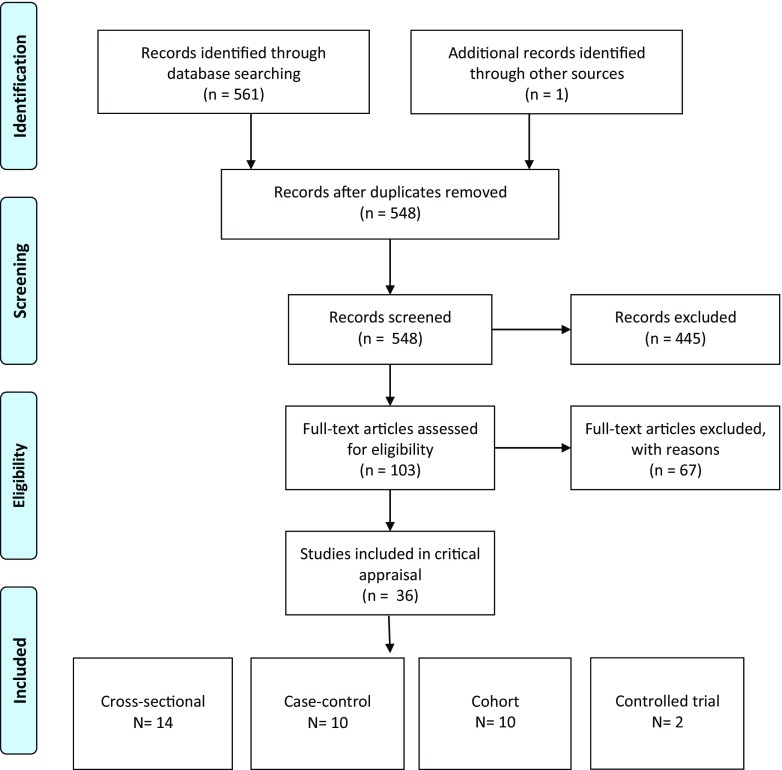
Flow chart

The search yielded a total of 561 articles and after adjusting for duplicates, 548 studies remained. Of these, 445 studies were excluded based on title and abstract. The full texts of 103 studies were assessed in more detail, and 67 were subsequently excluded. Supplementary list 1 provides an overview of articles that were excluded after full text screening, including the reasons of exclusion. After screening the reference lists of the remaining 35 articles, 1 study was added. The flowchart of the search is presented in Fig. 1. A total of 36 studies met the inclusion criteria and were processed for critical appraisal and data extraction. During the review process, 14 authors were contacted for further information, and 11 of them responded (see Acknowledgements).

### Study characteristics

An overview of the 36 included studies is presented in Table 1; 14 were cross-sectional studies, 10 were case-control studies, 10 were cohort studies, and 2 were (randomized) controlled trials. Most of the studies were in English; the article of Sumi et al. was in Japanese [65]. All papers, except for the Japanese study, were assessed by the authors SD and TB. The Japanese study was assessed by a native Japanese speaker with dental knowledge (EY, see Acknowledgements).

**Table 1:** Characteristics of the included the studies about gingival and periodontal disease, mucosal pathology, salivary flow, and oral hygiene in older people with dementia

Author(s), year, country	Design	Living environment	Participants with dementia (= *N*)	Mean age years (SD)	Controls (= *N*)	Mean age years (SD)	Dementia assessment	Method to measure oral hygiene and oral health
Adam et al. 2006 UK [36]	Cross-sectional	Nursing homes	81 MoD-SeD	80.8 (7.6)	54 ND or MiD	85.5 (7.6)	AMT	Debris Index by Greene and Vermillion
Chalmers et al. 2002 Australia [37]	Cohort	Community	116	< 79 years: 91 80 + years: 25	116	< 79 years: 91 80 + years: 25	MMSE	Plaque Index by Silness and Loe
Chalmers et al. 2003 Australia [38]	Cohort	Community	103	< 79 years: 82 80 + years: 21	113	< 79 years: 88 80 + years: 25	MMSE	Plaque Index by Silness and Loe, mucosal pathology
Chapman et al. 1991 Australia [39]	Cross-sectional	Non-institutionalized	85 AD	74.9	0	–	Not described	Presence of deposits
Chen et al. 2010 USA [40]	Cohort	Community and nursing homes	119	81.5 (9.2)	372	73.8 (10.7)	Chart	Presence of calculus/plaque/gingival bleeding
Chen et al. 2013a USA [41]	Cross-sectional	Community	51 community	79.3 (8.0)	0	–	Chart	Presence of calculus/plaque/gingival bleeding, assistance need oral care
		Assisted living	18 assisted living	80.9 (12.6)				
		Nursing homes	501 NHR	82.6 (9.6)				
Chen et al. 2013b USA [42]	Cross-sectional	Nursing homes	501	82.6 (9.6)	199	76.1 (13.9)	Chart	Presence of calculus/plaque/gingival bleeding
Chen et al. 2013c USA [43]	Cross-sectional	Community	46	79.3	138	71.6	Chart	Assistance need oral care
Chu et al. 2014 China [44]	Case-control	Community	59 MiD AD	79.8 (7.4)	59	79.8 (7.4)	Chart	Community Periodontal Index, Salivary flow
Cohen-Mansfield et al. 2002 USA [45]	Cross-sectional	Nursing homes	21	88.0	0	–	MMSE, MDS-COGS	Presence of gingivitis and periodontal disease
De Souza Rolim et al. 2014a Brazil [46]	Case-control	Community	29 mild AD	75.2 (6.7)	30	61.2 (11.2)	NINCDS-ADRDA for AD, MMSE	Gingival Bleeding Index, probing pocket depth, clinical attachment level, Plaque Index O’Leary
De Souza Rolim et al. 2014b Brazil [47]	Cohort	Community	29 mild AD	75.2 (6.7)	0	–	NINCDS-ADRDA for AD, MMSE	Gingival Bleeding Index, probing pocket depth, clinical attachment level, Plaque Index O’Leary,
Elsig et al. 2013 Switzerland [48]	Cross-sectional	Acute bed geriatric hospital	29	82.5 (6.3)	22	81.9 (6.5)	NPT, MMSE, CERAD, CDR	presence of visible dental plaque
Fjeld et al. 2014 Norway [49]	RCT	Nursing homes	159	85.5 (7.7)	43	88.5 (6.6)	Evaluated by physician	Simplified Oral Hygiene Index (OHI-S) of Greene and Vermillion, mouth dryness
Gil-Montoya et al. 2016a [50]	Case-control	Community	133 MoD-SeD	80.0 (7.5)	324	79.8 (8.3)	DSM-IVR, NINCDS-ADRDA	Plaque Index by Silness and Loe, Bleeding Index by Ainamo and Bay
Gil-Montoya et al. 2016b [51]	Cross sectional	Community	73 MiD 66 MoD 36 SeD	76.4 (7.5) 77.6 (7.3) 80.4 (6.5)	156	77.4 (6.9)	NINCDS–ADRDA	Drug-induced xerostomia
Hatipoglu et al. 2011 Turkey [52]	Prospective cohort	Nursing homes	31 AD	67.6 (9.1)	47	65.3 (7.0)	MMSE	Oral hygiene status, mucosal pathology
Hoben et al. 2016 Canada [53]	Cohort	Nursing homes	1606	85.0 (7.5)	1105	83.4 (10.5)	Chart	Presence of debris, presence of inflamed, swollen or bleeding gums, daily oral health care by staff
Hoeksema et al. 2016 Netherlands [54]	Case-control	Nursing homes	479	84.0 (7.0)	246 somatic	81.1 (8.0)	MMSE	Visual plaque according to the score of Mombelli
Ide et al. 2016 [55]	Cohort	Community	59 MiD-MoD	77.6 (8.6)	0	–	NINCDS–ADRDA	Presence of visible plaque, bleeding on probing, pocket depth, presence of moderate and severe periodontitis CDC/AAP criteria
Kossioni et al. 2012 Greece [56]	Case-control	Psychiatric hospital	27	76.5 (6.8)	0		DSM-IV	Presence of plaque or calculus, mucosal pathology
Kossioni et al. 2013 Greece [57]	Cross-sectional	Psychiatric hospital	23	76.3 (7.1)	0	–	Mentally ill, including dementia	Mucosal complaints
Leal et al. 2010 Brazil [58]	Case-control	Community	20 MiD with medication	69.6 (5.9)	20	68.3 (8.3)	NPT, CDR, MMSE	Mucosal pathology, salivary flow
Lee et al. 2013 USA [59]	Cross-sectional	Community	19 MiD	83.9 (7.9)	169	77.4 (5.8)	MCI, MiD: DSM-IV	Periodontal pocket depth, plaque Index (unspecified)
Philip et al. 2012 Australia [0^60^]	Cross-sectional	Institutionalized	84	85.7 (9.6)	102	84.3 (9.9)	Chart, ADLOH	Degree of gingival inflammation, Plaque Index by O’Leary
Ribeiro et al. 2012 Brazil [61]	Cross-sectional	Community	30	79.1 (5.6)	30	67.8 (5.5)	ICD-10, DSM-IV, MMSE, CDR	Oral Health Index by Greene and Vermillion
Ship et al. 1990 USA [62]	Case-control	Community	28	68.0 (10.0)	35	70.0 (10.0)	NINCDS-ADRDA, CT, MRI, PET, NPT	Salivary flow
Ship et al. 1994 USA [63]	Cohort	Community	21	64.0 (9.0)	21	65.0 (12.0)	NINCDS-ADRDA, CT, MRI, PET, PT	Change in gingival status, periodontal pocket depth, and salivary flow, but no seperate baseline data (Ship et al.1990)
Srisilapanan et al. 2013 Thailand [64]	Cross-sectional	Community–memory clinic	69	75.5 (7.0)	0	–	Chart, MMSE	Community Periodontal Index (CPI), Assistance need oral care
Sumi et al. 2012 Japan [65]	Cohort	Community–neurology clinic	10 AD	77.7 (5.9)	0	–	NINCDS-ADRDA, MMSE	Gingival Index Loe-Silness, Plaque Index by Quigley-Hein modified by Turesky
Syrjala et al. 2012 Finland [66]	Cross-sectional	Community	49 AD	84.8 (5.6)	278	81.4 (4.6)	DSM-III-R, DSM-IV, McKeith	Number of teeth with periodontal pockets ≥4 mm, presence of poor oral hygiene
			16 VaD	82.2 (4.7)				
			11 OD	85.3 (4.8)				
Warren 1997 USA [67]	Case-control	Community–geriatric assessment clinic	45 AD	81.6 (6.9)	133	80.3 (6.8)	MMSE, Chart, NT, Scans	Modification of Gingival Index by Silness and Loe, modification of Debris Index by Greene and Vermillion, mucosal pathology, xerostomia
			52 OD	81.4 (7.3)				
Zenthöfer et al. 2014 Germany [68]	Cohort	Long-term care homes	57	83.1 (10.6)	36	82.6 (9.0)	Chart	Periodontitis, Gingival Bleeding Index, CPITN, Dental Hygiene Index, Plaque Control Record by O’Leary
Zenthöfer et al. 2015 Germany [69]	Cohort	Long-term care homes	33	81.7 (9.0)	60	83.4(10.4)	chart	Gingival Bleeding Index, CPITN, Plaque Control Record by O’Leary, mucosal pathology
Zenthöfer etl al. 2016a Germany [70]	Case-control	Nursing homes	136	84.6 (8.1)	83	80.7 (9.8)	MMSE	Gingival Bleeding Index, community index of periodontal treatment needs
Zenthöfer etl al. 2016b Germany [71]	Controlled trial	Nursing homes	136	84.6 (8.1)	83	80.7 (9.8)	MMSE	Gingival Bleeding Index, community index of periodontal treatment needs

*AD* Alzheimer’s dementia, *AMT* abbreviated mental test, *CDC/AAP* Centre for Disease Control/American Academy of Periodontology, *CDR* Clinical Dementia Rating, *CPITN* community periodontal index of treatment needs, *CT* computer tomography, *DSM* Diagnostic and Statistical Manual of Mental Disorders, *ICD* International Classification of Diseases, *McKeith* consensus criteria presented by McKeith, *MMSE* Mini–Mental State Examination, *MRI* magnetic resonance imaging, *NHR *nursing home residents, *NINCD-ADRDA* National Institute of Neurological Disorders and Stroke Alzheimer’s Disease and Related Disorders Association, *NPT* Neuropsychological Testing, *NT* neurological testing, *OD* other dementia, *PET* positron emission tomography, *VaD* vascular dementia

Various dementia subtypes were reported in four studies [46, 61, 66, 67], and various dementia severities were reported in six of the studies [36, 46, 61, 66–68]. Five studies described the oral health of nursing home residents [53, 72–75], including people with dementia, but did not provide separate data for people with and without dementia.

### Critical appraisal

The results of the critical appraisal with the Newcastle-Ottawa scale are presented in Supplementary Table 1a–c. The total scores of the 34 articles appraised with the NOS ranged from 1 to 9, the mean score was 4.8 (SD 1.9), and the median was 5.0. Thirteen studies scored below and 12 studies scored above the median score. Hereafter, the separate NOS categories will be discussed. The DSM or ICD was used for the classification of the dementia diagnosis in half of the studies. The cases, i.e., older people with dementia, demonstrated good representativeness of the group of older persons with dementia in 24 (= 70.6%) of the 34 studies. The controls, i.e., older people without dementia, came from other sources than the cases in 12 (= 35.3%) of the 34 studies. For respectively 16 (= 47.1%) and 14 (= 41.2%) of the studies, age and gender were comparable between the groups with and without dementia. A standardized structured dental examination by a dentist was done in 29 (= 85.3%) of the studies. The non-response rate was described in only 2 (= 8.3%) of the 24 non-cohort studies. The follow-up period was longer than 3 months in 9 out of 10 (= 90.0%) cohort studies. The number of subjects lost to follow-up was described in 4 of 10 (= 40.0%) of the cohort studies.

The results of the critical appraisal of the two (randomized) controlled trials with the Delphi list are presented in Supplementary Table 2.

### Individual outcome variables

#### Gingival and periodontal disease

Table 2 and 3 show gingival and periodontal disease in older people with dementia in percentages and means. Gingival bleeding was absent in 0.0 to 9.4% of the participants with dementia [41, 64]. Consequently, most of the participants had gingival bleeding or inflammation [40, 42, 60]. More specifically, gingivitis was present in 13.6 to 38.9% [45, 46, 55], moderate periodontitis in 6.9 to 36.0%, and severe periodontitis in 11.9 to 24.5% of the participants with dementia [41, 46, 55, 75]. The mean percentage of the Gingival Bleeding Index was 46.0% in a study by De Souza Rolim [46] and 43.8–53.8% in the publications by Zenthöfer [68, 70, 76].

**Table 2:** Prevalence (in percentages) of gingival and periodontal disease in older people with dementia, compared with older people without dementia

Study	Dementia Number of participants Mean age (SD)	No dementia Number of participants Mean age (SD)	Gingival health outcome measure	Dementia Prevalence % (SD)	No Dementia Prevalence % (SD)
Chen et al. 2010	119	372	No calculus/plaque/gingival bleeding	0.9%	1.2%
	81.5 (9.2)	73.8 (10.7)	Mild to moderate calculus/plaque/gingival bleeding	67.9%	85.5%
			High calculus/plaque/gingival bleeding	31.3%	13.3%
Chen et al. 2013a	570 82.3	0	No calculus/plaque/gingival bleeding	Community 0% Assisted 8.3% NHR 0.3%	–
			Small to medium calculus/ plaque/gingival bleeding	Community 65.8% Assisted 66.7% NHR 59.2%	–
			High calculus/plaque/gingival bleeding	Community 34.2% Assisted 25.0% NHR 40.5%	–
Chen et al. 2013b	501	199	No calculus/plaque/gingival bleeding	0.3%	0.0%
	82.6 (9.6)	76.1 (13.9)	Mild to moderate calculus/plaque/gingival bleeding	59.2% Overall**	73.8%
			High calculus/plaque/gingival bleeding	40.4%	26.2%
Chu et al. 2014	59 MiD AD 79.8 (7.4)	59 79.8 (7.4)	Community Periodontal Index; pockets ≥ 3 mm	78.0%	74.0%
Cohen-Mansfield et al. 2002	21 88.0	0	Periodontal disease Gingivitis	44.4% 38.9%	–
De Souza Rolim et al. 2014a/b	29 mild AD	30 61.2 (11.2)	Gingivitis	31.0%	10.0%
	75.2 (6.7)		Moderate periodontitis	6.9%	10.0%
			Severe periodontitis	20.7%	6.7%
			Periodontal infection	58.6%**	26.7%
			Gingival Bleeding Index, mean % (SD)	46.0% (30.0)	–
Hoben et al. 2016	1606 85.0 (7.5)	1105 83.4 (10.5)	Presence of inflamed, swollen or bleeding gums (RAI-MDS 2.0)	0.8%	1.2%
Gil-Montoya et al. 2016a	133 MoD-SeD 80.0 (7.5)	324 79.8 (8.3)	Bleeding Index by Ainamo and Bay, mean % (SD)	67.5 (32.6)***	50.6 (34.2)
Hopcraft et al. 2012	105	170	Periodontal pocket depth 4 mm + periodontal pocket depth 6 mm+	36.0%	35.0%
				13.5%	7.0%
Ide et al. 2016	59 MiD-MoD 77.6 (8.6)	0	Probing sites deeper than 3 mm Bleeding on probing Periodontitis according to CDC/AAP criteria: -moderate periodontitis -severe periodontitis	6.7% 13.6% 37.3% 25.4% 11.9%	–
Philip et al.2012	84 85.7 (9.6)	102 84.3 (9.9)	Gingival inflammation (% of teeth with erythema): Minimal (0–10%) Light (20–40%) Moderate (50–70%) Heavy (80–100%)	13.0% 21.0% 56.5% 13.0%	22.5% 36.3% 32.5% 6.8%
Srisilapanan et al. 2013	69 75.5 (7.0)	0	Community Periodontal Index (CPI), highest score: Normal Bleeding Calculus Pocket depth 4-5 mm Pocket depth ≥ 6 mm	9.4% 1.9% 34.0% 30.2% 24.5%	– – – – –
Zenthöfer et al. 2014	57 83.1 (10.6)	36 82.6 (10.6)	Periodontitis Gingival Bleeding Index, mean % (SD)	100.0%*** 43.8% (23.7)	73.9% 40.9% (25.1)
Zenthöfer et al. 2015	33 81.7 (9.0)	60 83.4 (10.4)	Gingival Bleeding Index, mean % (SD)	52.1% (29.1)*	38.1% (20.1)
Zenthofer et al. 2016 a	136 84.6 (8.1)	83 80.7 (9.8)	Gingival Bleeding Index	53.8% (27.6)	48.8 (28.9)

*p ≤ .05, **p ≤ .01, ***p ≤ .001, *NHR* nursing home residents, *CDC/AAP* Centre for Disease Control/American Academy of Periodontology

**Table 3:** Indices (in means) of gingival and periodontal disease of older people with dementia, compared with older people without dementia

Study	Dementia Number of participants Mean age (SD)	No dementia Number of participants Mean age (SD)	Gingival health outcome measure	Dementia Mean (SD)	No Dementia Mean (SD)
De Souza Rolim et al. 2014a	29 mild AD	30 61.2 (11.2)	Probing pocket depth (in mm)	1.6 mm (0.7)	–
	75.2 (6.7)		Clinical attachment level (in mm)	2.9 mm (1.3)	–
Ide et al. 2016	59 MiD-MoD 77.6 (8.6)	0	Probing depth (in mm)	2.5 mm (0.4)	–
Lee et al. 2013	19 MiD 83.9 (7.9)	169 77.4 (5.8)	Periodontal pocket depth (in mm)	MiD 1.4 mm (1.1)	1.5 mm (1.2)
Sumi et al. 2012	10 77.7 (5.9)	0	Gingival Index Loe-Silness (index score)	1.2	–
Syrjala et al.2012	49 AD 84.8 (5.6)	278 81.4 (4.6)	Number of teeth with periodontal pockets ≥ 4 mm	AD 2.8 (3.3)	2.9 (3.8)
	16 VaD 82.2 (4.7)			VaD 2.8 (3.8)	
	11 OD 85.3 (4.8)			OD 1.7 (1.5)	
Warren et al. 1997	45 AD 81.6 (6.9)	133 80.3 (6.8)	Modified version of the Gingival Index by Silness and Loe (index score)	AD 1.1 (0.8)*	0.7 (0.6)
	52 OD 81.4 (7.3)			OD 0.9 (0.7)	
Zenthöfer et al. 2014	57	36 82.6 (10.6)	Community periodontal index of treatment needs (index score)	3.4 (0.5)***	2.8 (0.6)
	83.1 (10.6)				
Zenthöfer et al. 2015	33 81.7 (9.0)	60 83.4 (10.4)	Community periodontal index of treatment needs (index score)	3.3 (0.6)	3.1 (0.6)
Zenthofer et al. 2016 a, b	136 84.6 (8.1)	83 80.7 (9.8)	Community periodontal index of treatment needs (index score)	3.1 (0.7)***	2.7 (0.6)

*p ≤ .05, **p ≤ .01, ***p ≤ .001, *AD* Alzheimer’s dementia, *Mid* mild dementia, *ND* no dementia, *OD* other dementia’s, *VaD* vascular dementia

When examining studies that compared gingival and periodontal disease of participants with and without dementia, seven studies showed no significant differences [44, 53, 59, 60, 66, 70, 75] and six studies showed significantly more (severe) periodontal disease in older people with dementia [42, 46, 50, 67, 68, 70]. De Souza Rolim et al. reported significantly more periodontal infection in participants with dementia (58.6%) than in participants without dementia (26.7%) [46]. Zenthöfer et al. found significantly more periodontitis in participants with dementia (100.0%) than in participants without dementia (73.9%) [68]. Furthermore, the community periodontal index of treatment needs was significantly higher in participants with dementia (3.1–3.4) than without dementia (2.7–2.8) [68, 70]. When specifically looking at nursing home residents, a significantly higher percentage of participants with dementia had a high amount of calculus, plaque, or gingival bleeding (40.4%), when compared to people without dementia (26.2%). A study by Warren et al. found that the Gingival Index was significantly higher in people with Alzheimer’s disease (1.1) than in people without dementia (0.7), while people with dementia other than Alzheimer’s disease scored not significantly different (0.9) from people with Alzheimer’s disease and people without dementia [67].

#### Oral hygiene and assistance need

Table 4 and 5 show the oral hygiene measures in percentages and means in older people with dementia, compared with older people without dementia. Studies including the Plaque Index by O’Leary found a mean percentage of 63.4 to 90.1% in participants with dementia [46, 60, 68, 76]. Studies using the indices by Greene and Vermillion found a Debris Index of 2.1, a Calculus Index of 2.0, and an Oral Hygiene Index of 4.5 in participants with dementia [36, 49, 61]. The Plaque Index by Silness and Loe was 0.7 in a study by Chalmers et al. and 2.5 in a study by Gil Montoya et al. [38, 50]. Sumi et al. reported a Plaque Index by Quigley and Hein (modified by Turesky) of 1.6 in people with dementia [65].

**Table 4:** Oral hygiene measures (in percentages) of older people with dementia, compared with older people without dementia

Study	Dementia Number of participants Mean age (SD)	No dementia Number of participants Mean age (SD)	Oral hygiene outcome measure	Dementia Prevalence % (SD)	No dementia Prevalence % (SD)
Chapman et al. 1991	85 AD 74.9	–	Soft deposits Hard deposits Unsatisfactory level of oral hygiene	70.0% 60.0% 90.0%	–
De Souza Rolim et al. 2014 a, b	29 mild AD 75.2 (6.7)	30 61.2 (11.2)	Plaque Index by O’Leary, mean % (SD)	73.6% (5.7)	–
Elsig et al. 2013	29 82.5 (6.3)	22 81.9 (6.5)	Presence of visible dental plaque	100.0%**	36.0%
Hatipoglu et al. 2011	31 AD 67.6 (9.1)	47 65.3 (7.0)	Good oral hygiene Fair oral hygiene Poor oral hygiene	3.2% 29.0% 67.7%	19.1% 31.9% 48.9%
Hoben et al. 2016	1606 85.0 (7.5)	1105 83.4 (10.5)	Presence of debris (RAI-MDS 2.0)	9.8%	11.4%
Hoeksema et al. 2016	103 80.8 (7.5)	49 somatic 78.1 (7.9)	Poor oral hygiene (Mombelli score 2 or 3)	72.8%	77.6%
Hopcraft et al. 2012	105	170	Thin band of visual plaque < 1/3 tooth with visual plaque > 1/3 tooth with visual plaque	26.3% 43.4% 30.3%	36.5% 38.4% 25.2%
Ide et al. 2016	59 MiD-MoD 77.6 (8.6)	0	Detectable plaque: -without a dental probe -with a dental probe	89.0% (12.5) 19.9% (11.8) 69.1% (20.6)	–
Kossioni et al. 2012	27 76.5 (6.8)	–	Presence of plaque or calculus	80.0%	–
Philip et al. 2012	84 85.7 (9.6)	102 84.3 (9.9)	Plaque Index by O’Leary, mean % (SD)	63.4% (35.7)	54.5 (35.7)
Syrjala et al. 2012	49 AD 84.8 (5.6) 16 VaD 82.2 (4.7) 11 OD 85.3 (4.8)	278 81.4 (4.6)	Presence of poor oral hygiene	AD 77.8% VaD 60.0% OD 66.7%	36.6%
Zenthöfer et al. 2014	57 83.1 (10.6)	36 82.6 (10.6)	Plaque Control Record by O’Leary, mean % (SD)	90.1% (13.1)**	73.3% (25.1)
Zenthöfer et al. 2015	33 81.7 (9.0)	60 83.4 (10.4)	Plaque Control Record by O’Leary, mean % (SD)	89.3% (12.6)	80.3% (23.0)

*p ≤ .05, **p ≤ .01, ***p ≤ .001, *AD* Alzheimer’s dementia, *OD* other dementia’s, *VaD* vascular dementia

**Table 5:** Oral hygiene indices (in means) of older people with dementia compared with older people without dementia

Study	Dementia Number of participants Mean age (SD)	No dementia Number of participants Mean age (SD)	Oral hygiene outcome measure	Dementia Mean (SD)	No dementia Mean (SD)
Adam et al. 2006	81 MoD-SeD 80.8 (7.6)	54 ND-MiD 85.5 (7.6)	Debris Index by Greene and Vermillion	MoD/SeD 2.1 (0.7)	ND/MiD 1.3 (0.6)
			Calculus Index by Greene and Vermillion	MoD/SeD 2.0 (0.8)	ND/MiD 1.3 (0.6)
Chalmers et al. 2003	116 < 79 years: 91 80 + years: 25	116 < 79 years: 91 80 + years: 25	Plaque Index by Silness and Loe	0.7 (mv)	0.6 (mv)
Gil-Montoya et al. 2016	133 MoD-SeD 80.0 (7.5)	324 79.8 (8.3)	Plaque Index by Silness and Loe	2.5 (0.6)***	1.6 (0.9)
Hoeksema et al. 2016	103 80.8 (7.5)	49 somatic 78.1 (7.9)	Visual plaque score Mombelli	2.1 (0.9)	2.3 (0.9)
Lee et al. 2013	19 83.9 (7.9)	169 77.4 (5.8)	Plaque Index (unspecified)	MiD 0.9 (0.7)	0.5 (0.6)
Ribeiro et al. 2012	30 79.1 (5.6)	30 67.8 (5.5)	Oral Hygiene Index by Greene and Vermillion	4.5 (1.7–10.0)**	2.2 (0.3–8.0)
Sumi et al. 2012	10 77.7 (5.9)	0	Plaque Index by Quigley-Hein (modified by Turesky)	1.6	–
Warren et al. 1997	45 AD 81.6 (6.9) 52 OD 81.4 (7.3)	133 80.3 (6.8)	Modification of the Debris Index by Greene and Vermillion	AD 1.0 (0.8) OD 1.0 (0.8) MiD 1.0 (0.7) MoD-SeD 1.1 (0.9)*	0.8 (0.6)

*p ≤ .05, **p ≤ .01, ***p ≤ .001, *AD* Alzheimer’s dementia, *MiD* mild dementia, *MoD* moderate dementia, *ND* no dementia, *OD* other dementia’s, *SeD* severe dementia, *VaD* vascular dementia

When examining studies that compared oral hygiene in participants with and without dementia, nine studies found no significant differences [36, 38, 52, 53, 59, 60, 66, 75, 76] and five studies found significantly more plaque in people with dementia [48, 50, 61, 67, 68]. Elsig et al. reported 100.0% visible plaque in participants with dementia and 36.0% in those without dementia [48]. Furthermore, a significantly higher O’Leary Plaque Index was found in participants with dementia (90.1%), compared to participants without dementia (73.3%) [68]. In addition, a significantly higher Oral Hygiene Index by Greene and Vermillion was found in participants with dementia (4.5) than in participants without dementia (2.2) [61]. A recent study found significantly more plaque with the Plaque Index of Silness and Loe in cases (2.5) than controls (1.6) [50].

Warren examined the Debris Index for dementia subtypes and severities and found no significant differences between dementia subtypes and controls, but found a significantly higher Debris Index in people with moderate to severe dementia, compared to people without dementia [67].

Studies about assistance need for oral care (Supplementary Table 3) reported a need of 21% and higher for both cleaning teeth and dentures in participants with dementia [38, 41, 42, 60, 64, 72, 75]. Chalmers et al. reported a significantly higher need for assistance with oral hygiene care with an increasing severity of cognitive impairment [72]. In this study, the assistance need was 57.2% for cleaning teeth and 97.3% for cleaning dentures in people with moderate dementia, and 100.0% for cleaning teeth as well as dentures in people with severe dementia [72]. In all except 1 of 7 the comparing studies the assistance need for oral care or cleaning teeth and dentures was significantly higher in people with dementia than in those without [41, 42, 60, 72, 75].

#### Oral pathology and oral dryness

Candidiasis (Supplementary Table 4) was present in 3.6 to 30.0% of the cases, i.e., older people with dementia, and 0.0 to 5.0% of the controls, i.e., older people without dementia [44, 46, 47, 58]. The study by De Souza Rolim et al. (2014) found significantly more candidiasis in cases than in controls. Stomatitis was present in 18.1 to 59.1% of the cases and 0.0 to 7.4% of the controls [38, 52, 56]. The only study that compared stomatitis in cases and controls found significantly more stomatitis in cases than in controls [52].

Xerostomia (Supplementary Table 4), i.e., a subjective feeling of a dry mouth, was present in 9.1 to 45.0% of the cases and 8.4 to 20.0% of the controls [57, 58, 67, ]. Warren et al. found significantly more xerostomia in people with dementia subtypes other than Alzheimer’s disease (22.0%) than in people without dementia (8.4%) in their clinician assessment of xerostomia. In addition, xerostomia was found in 9.1% of the people with Alzheimer’s disease in this study [67]. A recent study found significantly more drug-induced xerostomia in cases (68.5–72.2%) than in controls (36.5%) [51].

In addition, various studies indicated that people without dementia have significantly more unstimulated salivary flow (Supplementary Table 5) than people with dementia [39, 44, 58]. One study indicated that people without dementia have more stimulated submandibular flow than Alzheimer’s disease [39]. The study of Leal et al. (2010) showed the buffering capacity is higher in people without dementia and without medication than in people with dementia and medication [58].

## Discussion

The main aim of this study was to provide a comprehensive overview with critical appraisal of studies concerning the health of oral soft tissues and oral hygiene in older people with dementia. The additional aim was to compare oral health of older people with and without dementia. The studies included in this review suggest that older people with dementia have much plaque and many oral health problems related to oral soft tissues, such as gingival bleeding, periodontal pockets, stomatitis, mucosal lesions, and reduced salivary flow.

While oral health in people with dementia is poor [42, 66, 68], the subtype of dementia, e.g., Alzheimer’s disease, vascular dementia, does not seem to be an essential determinant of oral health [38, 66, 67]. However, the severity of cognitive decline does seem to play a role in the oral health of older people with dementia, with more plaque and oral disease in people with more cognitive decline [36, 38, 66, 67, 72]. An exception to this finding is the study of Srisilapanan et al. [64], which was explained by the fact that people visiting the memory clinic in this study also got dental treatment with every visit. The people included in this study had better access to oral care compared to the general population. In addition, living environment, e.g., nursing home, community, might play a role in the oral health of older people with dementia [61, 72]. Some studies found no significant differences between living environments, but a poor oral health in people with dementia, regardless of residency [38, 41]. Some studies found more problems in nursing home residents compared to community living elderly [61, 72]. This can be explained by a higher degree of cognitive and functional impairment in the nursing home population [41].

The deterioration of cognitive functions, such as executive functioning, working memory, attention, and apraxia, complicates the ability to perform oral care in people with dementia [42], which results in more plaque [60, 68]. Furthermore, functional changes in dementia, like declined hand grip strength and motor skills, can complicate oral care [52, 61]. Dental plaque is the primary cause of gingivitis and subsequently periodontitis [77]. Therefore, with a high amount of plaque in older people with dementia in the evaluated articles, a high amount of gingivitis and periodontitis could be expected. Periodontitis has been associated with multiple systemic health conditions (mainly diabetes mellitus type 2 and cardiovascular disease) [4]. Therefore, treatment of periodontitis is important to reduce systemic health risks.

Recently, the association between oral health and cognitive decline was systematically reviewed. The authors concluded that the association was still unclear [18, 19].

Within the included papers, salivary function appeared to be intact in the healthy aging sample. However, objective (hyposalivation) or subjective (xerostomia) dry mouth is more likely to be present in older people with medication use, history of radiotherapy in the head and neck region, and autoimmune disease, such as Sjögren syndrome [5, 11, 78]. In addition, people with Alzheimer’s disease have significantly lower stimulated submandibular and unstimulated salivary flow rate [39, 44, 58]. This might be explained by neuropathological changes characteristic for Alzheimer’s disease, leading to changes in the autonomic nervous system [39, 44].

## Critical appraisal

The NOS comprises five categories concerning a control group, which means studies without control group consequently receive a lower score. Six of the 14 included studies with cross-sectional design did not have a control group and therefore had a NOS score below the median score of 5. Consequently, for the studies without a control group, it was not possible to compare older people with and without dementia. Furthermore, most of the included studies did not describe the non-response rate and consequently lacked a point on the NOS for this category. An issue worth mentioning, is that for the data-extraction of this review only baseline data was used, while the NOS also includes two items concerning follow-up studies for cohort studies. These two follow-up items in the 10 included cohort studies are not relevant for this review, but might be of interest for further appraisal of the studies.

## Strengths and limitations

The main strengths of this review are the critical appraisal of the articles, the summary tables of the dementia and oral health variables, and the involvement of a multidisciplinary team. Concerning the critical appraisal, most studies demonstrated good representativeness of older people with dementia, and almost all included studies followed a standardized structured dental examination by a dentist. The involvement of the multidisciplinary team critically evaluated the dental, neuropsychological, medical, ethical, and methodological aspects of this study.

Limitations of this review are the following: the included studies used a broad range of outcome measures, most studies had a cross-sectional design, and the number of studies with a NOS score below 5 was considerable. However, if these studies would have been excluded, the tendency of the results for the group of older people with dementia would have remained the same. It was therefore decided to keep the studies with a NOS score below 5 in the overview of the results. In addition, the methodological and clinical heterogeneity between the studies was considered too large to perform a meta-analysis. Furthermore, many of the included studies did not use a formal dementia diagnosis or diagnostic instruments, but screening instruments for cognition. In these studies, it might be more appropriate to address to the participants as people with cognitive impairment instead of people with dementia. In addition, the non-response rate was rarely described, which complicates the assessment of potential selection-bias.

## Implications and clinical suggestions

In order to improve the oral health of older people with dementia, oral health assessment tools, oral hygiene care strategies, and guidelines should be used [79–83]. To maintain good oral health, daily removal of dental plaque by brushing the teeth is essential [84]. Therefore, oral hygiene care for dependent people should be in the daily activities of care [81, 85] and oral health care education might improve the knowledge and attitude of caretakers [86]. Although providing oral care and dental treatment can be complicated by challenging behavior, strategies that approach it as threat perception might help the well-being of people with dementia [87]. Regular assessment of the oral health should take place by caretakers, as well as dentists [81]. An oral health assessment tool can be used to identify risk factors and should consist of intra-oral examination, observation of behavior, and (if possible) an evaluation of the client perception of treatment need [82, 88]. For oral care, treatment planning, and behavioral management for people with dementia, the level of cognitive impairment and cooperation of the patient, as well as the input from the multi-disciplinary team of health care professionals, and formal and informal caretakers should be taken into account [17, 89].

## Future research

For future research, it is suggested to use a formal diagnosis for dementia [90]. If neuropsychological testing is no longer possible, a short cognitive screening instrument, such as the Mini–Mental State Examination (MMSE), can be used to get an impression of the level of cognitive functioning [91]. However, a low score on the MMSE is only an indication of cognitive impairment and does not replace the diagnostic examination required for a dementia diagnosis [92].

For the oral health assessment in older people with dementia, an international, standardized method can be useful. Although the manual WHO Oral Health Survey Basic Methods provides no information on assessing the oral health status of older people with dementia [93], guidelines for oral health care for institutionalized older people do exist [81, 82, 94].

## Conclusion

The studies included in the current systematic review suggest that older people with dementia have high levels of plaque and many oral health problems related to oral soft tissues, such as gingival bleeding, periodontal pockets, stomatitis, mucosal lesions, and reduced salivary flow.

The oral hygiene and oral health of older people with dementia should be improved. This could be achieved by oral care education of formal and informal caregivers, the use of oral health screening tools, and regular professional dental care for people with dementia.

## Electronic supplementary material

ESM 1 (DOCX 28 kb)

ESM 2 (DOCX 81 kb)
