# Supplementary material for: Oral hygiene and oral health in older people with dementia: a comprehensive review with focus on oral soft tissues
Source: Clin Oral Investig. 2017 Nov 15;22(1):93–108. doi: 10.1007/s00784-017-2264-2 (PMC5748411; doi:10.1007/s00784-017-2264-2)
Supplement: Supplementary file 1 — (DOCX 28 kb) [file 784_2017_2264_MOESM1_ESM.docx]

**Supplementary List of Excluded Articles**

Clinical update

1. Dean E (2016) Oral health for adults in care homes. Nurs Stand 31:15. doi: 10.7748/ns.31.2.15.s16

No dementia group with specific oral health data

2. Arai K, Sumi Y, Uematsu H, Miura H (2003) Association between dental health behaviours, mental/physical function and self-feeding ability among the elderly: a cross-sectional survey. Gerodontology 20:78–83. doi: 10.1111/j.1741-2358.2003.00078.x

3. Chalmers J, King P, Spencer A, et al (2005) The Oral Health Assessment Tool ? Validity and reliability. Aust Dent J 50:191–199. doi: 10.1111/j.1834-7819.2005.tb00360.x

4. Comfort AO, King T, Moveni M, et al (2004) Dental health of Fiji institutionalized elderly (2003). Pac Health Dialog 11:38–43 6p.

5. Gil-Montoya JA, Ponce G, S?nchez Lara I, et al (2013) Association of the oral health impact profile with malnutrition risk in Spanish elders. Arch Gerontol Geriatr 57:398–402. doi: 10.1016/j.archger.2013.05.002

6. Maxwell NI, Shah S, Dooley D, et al (2014) Oral Health among Residents of Publicly Supported Housing in Boston. J Urban Heal 91:809–821. doi: 10.1007/s11524-013-9845-4

7. Morishita S, Watanabe Y, Ohara Y, et al (2016) Factors associated with older adults’ need for oral hygiene management by dental professionals. Geriatr Gerontol Int 16:956–962. doi: 10.1111/ggi.12585

8. Mozafari PM, Dalirsani Z, Delavarian Z, et al (2012) Prevalence of oral mucosal lesions in institutionalized elderly people in Mashhad, Northeast Iran. Gerodontology 29:e930–e934. doi: 10.1111/j.1741-2358.2011.00588.x

9. Nishiyama Y (2005) [Changes of general and oral health status of elderly patients receiving home-visit dental services]. Kokubyo Gakkai Zasshi 72:172–82.

10. Stubbs C, Riordan P (2002) Dental screening of older adults living in residential aged care facilities in Perth. Aust Dent J 47:321–326. doi: 10.1111/j.1834-7819.2002.tb00545.x

11. Teng P-R, Lin M-J, Yeh L-L (2016) Utilization of dental care among patients with severe mental illness: a study of a National Health Insurance database. BMC Oral Health 16:87. doi: 10.1186/s12903-016-0280-2

12. Walker RJ, Kiyak HA (2007) The impact of providing dental services to frail older adults: Perceptions of elders in adult day health centers*. Spec Care Dent 27:139–143. doi: 10.1111/j.1754-4505.2007.tb00336.x

*Focus on relation oral health and cognition*

13. Arrivé E, Letenneur L, Matharan F, et al (2012) Oral health condition of French elderly and risk of dementia: a longitudinal cohort study. Community Dent Oral Epidemiol 40:230–238. doi: 10.1111/j.1600-0528.2011.00650.x

14. Batty G-D, Li Q, Huxley R, et al (2013) Oral disease in relation to future risk of dementia and cognitive decline: Prospective cohort study based on the Action in Diabetes and Vascular Disease: Preterax and Diamicron Modified-Release Controlled Evaluation (ADVANCE) trial. Eur Psychiatry 28:49–52. doi: 10.1016/j.eurpsy.2011.07.005

15. Naorungroj S, Slade GD, Beck JD, et al (2013) Cognitive Decline and Oral Health in Middle-aged Adults in the ARIC Study. J Dent Res 92:795–801. doi: 10.1177/0022034513497960

16. Noble JM, Borrell LN, Papapanou PN, et al (2009) Periodontitis is associated with cognitive impairment among older adults: analysis of NHANES-III. J Neurol Neurosurg Psychiatry 80:1206–1211. doi: 10.1136/jnnp.2009.174029

17. Nilsson H, Berglund J, Renvert S (2014) Tooth loss and cognitive functions among older adults. Acta Odontol Scand 72:639–644. doi: 10.3109/00016357.2014.882983

18. Paganini-Hill A, White SC, Atchison KA (2012) Dentition, Dental Health Habits, and Dementia: The Leisure World Cohort Study. J Am Geriatr Soc 60:1556–1563. doi: 10.1111/j.1532-5415.2012.04064.x

19. Rai B, Kaur J, Anand SC (2012) Possible relationship between periodontitis and dementia in a North Indian old age population: a pilot study. Gerodontology 29:e200–e205. doi: 10.1111/j.1741-2358.2010.00441.x

20. Shimazaki Y, Soh I, Saito T, et al (2001) Influence of Dentition Status on Physical Disability, Mental Impairment, and Mortality in Institutionalized Elderly People. J Dent Res 80:340–345. doi: 10.1177/00220345010800010801

21. Stein PS, Desrosiers M, Donegan SJ, et al (2007) Tooth loss, dementia and neuropathology in the Nun Study. J Am Dent Assoc 138:1314–1322.

22. Stein PS, Kryscio RJ, Desrosiers M, et al (2010) Tooth Loss, Apolipoprotein E, and Decline in Delayed Word Recall. J Dent Res 89:473–477. doi: 10.1177/0022034509357881

23. Stewart R, Stenman U, Hakeberg M, et al (2015) Associations Between Oral Health and Risk of Dementia in a 37-Year Follow-Up Study: The Prospective Population Study of Women in Gothenburg. J Am Geriatr Soc 63:100–105. doi: 10.1111/jgs.13194

24. Watanabe I, Kuriyama N, Miyatani F, et al (2016) Oral Cnm-positive Streptococcus Mutans Expressing Collagen Binding Activity is a Risk Factor for Cerebral Microbleeds and Cognitive Impairment. Sci Rep 6:38561. doi: 10.1038/srep38561

25. Yamamoto T, Kondo K, Hirai H, et al (2012) Association Between Self-Reported Dental Health Status and Onset of Dementia. Psychosom Med 74:241–248. doi: 10.1097/PSY.0b013e318246dffb

No useable oral health data

26. Akiyama S, Imanishi H, Yasufuku Y, et al (1993) Dental findings of the elderly with or without senile dementia at a special nursing home. J Osaka Univ Dent Sch 33:21–6.

27. Blanco VL, Levy SM, Ettinger RL, et al (1997) Challenges in geriatric oral health research methodology concerning caregivers of cognitively impaired elderly adults. Spec Care Dent 17:129–132. doi: 10.1111/j.1754-4505.1997.tb00882.x

28. Cockburn AF, Dehlin JM, Ngan T, et al (2012) High throughput DNA sequencing to detect differences in the subgingival plaque microbiome in elderly subjects with and without dementia. Investig Genet 3:19. doi: 10.1186/2041-2223-3-19

29. Ericsson I, Aronsson K, Cedersund E, et al (2009) The meaning of oral health-related quality of life for elderly persons with dementia. Acta Odontol Scand 67:212–221. doi: 10.1080/00016350902855296

30. Fujihara I, Sadamori S, Abekura H, Akagawa Y (2013) Relationship between behavioral and psychological symptoms of dementia and oral health status in the elderly with vascular dementia. Gerodontology 30:157–61. doi: 10.1111/j.1741-2358.2012.00664.x

31. Holm B, Söderhamn O (2003) Factors associated with nutritional status in a group of people in an early stage of dementia. Clin Nutr 22:385–9. doi: 10.1016/S0261-5614(03)00035-9

32. Horn VJ, Hodge WC, Treuer JP (1994) Dental condition and weight loss in institutionalized demented patients. Spec Care Dent 14:108–111. doi: 10.1111/j.1754-4505.1994.tb01114.x

33. Hugo FN, Hilgert JB, Bertuzzi D, et al (2007) Oral health behaviour and socio-demographic profile of subjects with Alzheimer’s disease as reported by their family caregivers. Gerodontology 24:36–40. doi: 10.1111/j.1741-2358.2007.00149.x

34. Kamer AR, Craig RG, Pirraglia E, et al (2010) TNF-α and antibodies to periodontal bacteria discriminate between Alzheimer’s disease patients and normal subjects. 216:92–97. doi: 10.1016/j.jneuroim.2009.08.013.TNF-

35. Katsoulis J, Huber S, Zumsteg P, et al (2009) [Gerodontic consultation service for hospitalized geriatric patients: diagnosis and therapy (II)]. Schweizer Monatsschrift fur Zahnmedizin = Rev Mens suisse d’odonto-stomatologie = Riv Mens Svizz di Odontol e Stomatol 119:688–94.

36. Kersten H, Wyller TB, Molden E (2014) Association between inherited CYP2D6/2C19 phenotypes and anticholinergic measures in elderly patients using anticholinergic drugs. Ther Drug Monit 36:125–30. doi: 10.1097/FTD.0b013e31829da990

37. Kobayashi N, Soga Y, Maekawa K, et al (2017) Prevalence of oral health-related conditions that could trigger accidents for patients with moderate-to-severe dementia. Gerodontology 34:129–134. doi: 10.1111/ger.12235

38. Kwak YT, Han I-W, Lee PH, et al (2009) Associated conditions and clinical significance of awake bruxism. Geriatr Gerontol Int 9:382–90. doi: 10.1111/j.1447-0594.2009.00538.x

39. Lee KH, Wu B, Plassman BL (2015) Dental care utilization among older adults with cognitive impairment in the USA. Geriatr Gerontol Int 15:255–60. doi: 10.1111/ggi.12264

40. Li H, Takeshita T, Furuta M, et al (2012) Molecular characterization of fungal populations on the tongue dorsum of institutionalized elderly adults. Oral Dis 18:771–7. doi: 10.1111/j.1601-0825.2012.01944.x

41. Lin CY, Jones DB, Godwin K, et al (1999) Oral health assessment by nursing staff of Alzheimer’s patients in a long-term-care facility. Spec Care Dent 19:64–71. doi: 10.1111/j.1754-4505.1999.tb01370.x

42. Machado MC, Lopes GH, Marchini L (2012) Oral health of Alzheimer’s patients in São José dos Campos, Brazil. Geriatr Gerontol Int 12:265–70. doi: 10.1111/j.1447-0594.2011.00763.x

43. Ní Chróinín D, Montalto A, Jahromi S, et al (2016) Oral Health Status Is Associated with Common Medical Comorbidities in Older Hospital Inpatients. J Am Geriatr Soc 64:1696–700. doi: 10.1111/jgs.14247

44. Nitschke I, Müller F, Hopfenmüller W (2001) The uptake of dental services by elderly Germans. Gerodontology 18:114–20. doi: 10.1111/j.1741-2358.2001.00114.x

45. Noble JM, Scarmeas N, Celenti RS, et al (2014) Serum IgG antibody levels to periodontal microbiota are associated with incident Alzheimer disease. PLoS One 9:e114959. doi: 10.1371/journal.pone.0114959

46. Sadamori S, Hayashi S, Hamada T (2008) The relationships between oral status, physical and mental health, nutritional status and diet type in elderly Japanese women with dementia. Gerodontology 25:205–9. doi: 10.1111/j.1741-2358.2008.00224.x

47. Sadamori S, Hayashi S, Fujihara I, et al (2012) Nutritional status and oral status of the elderly with dementia: a 2-year study. Gerodontology 29:e756-60. doi: 10.1111/j.1741-2358.2011.00555.x

48. Suzuki K, Nomura T, Sakurai M, et al (2005) Relationship between number of present teeth and nutritional intake in institutionalized elderly. Bull Tokyo Dent Coll 46:135–43.

Included in review hard tissues, but not suitable for review oral hygiene and soft tissues

49. Bomfim FMS, Chiari BM, Roque FP (2013) Factors associated to suggestive signs of oropharyngeal dysphagia in institutionalized elderly women. CoDAS 25:154–63. doi: 10.1590/S2317-17822013000200011

50. Chalmers JM, Hodge C, Fuss JM, et al (2002) The prevalence and experience of oral diseases in Adelaide nursing home residents. Aust Dent J 47:123–30. doi: 10.1111/j.1834-7819.2002.tb00315.x

51. Chalmers J, Carter K, Spencer A (2004) Oral health of Adelaide nursing home residents: longitudinal study. Australas J Ageing 23:63–70. doi: 10.1111/j.1741-6612.2004.00019.x

52. Chalmers JM, Carter KD, Spencer AJ (2005) Caries incidence and increments in Adelaide nursing home residents. Spec Care Dentist 25:96–105. doi: 10.1111/j.1754-4505.2005.tb01418.x

53. Chu CH, Ng A, Chau AMH, Lo ECM (2015) Oral health status of elderly chinese with dementia in Hong Kong. Oral Health Prev Dent 13:51–7. doi: 10.3290/j.ohpd.a32343

54. Del Brutto OH, Gardener H, Del Brutto VJ, et al (2014) Edentulism Associates with Worse Cognitive Performance in Community-Dwelling Elders in Rural Ecuador: Results of the Atahualpa Project. J Community Health 39:1097–1100. doi: 10.1007/s10900-014-9857-3

55. Ellefsen B, Holm-Pedersen P, Morse DE, et al (2008) Caries prevalence in older persons with and without dementia. J Am Geriatr Soc 56:59–67. doi: 10.1111/j.1532-5415.2007.01495.x

56. Ellefsen B, Holm-Pedersen P, Morse DE, et al (2009) Assessing caries increments in elderly patients with and without dementia: a one-year follow-up study. JADA 140:1392–1400. doi: 10.1002/pbc.24544

57. Ellefsen BS, Morse DE, Waldemar G, Holm-Pedersen P (2012) Indicators for root caries in Danish persons with recently diagnosed Alzheimer’s disease. Gerodontology 29:194–202. doi: 10.1111/j.1741-2358.2011.00560.x

58. Eshkoor SA, Hamid TA, Nudin SSH, Mun CY (2014) Association between dentures and the rate of falls in dementia. Med Devices (Auckl) 7:225–30. doi: 10.2147/MDER.S63220

59. Furuta M, Komiya-Nonaka M, Akifusa S, et al (2013) Interrelationship of oral health status, swallowing function, nutritional status, and cognitive ability with activities of daily living in Japanese elderly people receiving home care services due to physical disabilities. Community Dent Oral Epidemiol 41:173–81. doi: 10.1111/cdoe.12000

60. Hopcraft MS, Morgan M V, Satur JG, Wright FAC (2012) Edentulism and dental caries in Victorian nursing homes. Gerodontology 29:e512-9. doi: 10.1111/j.1741-2358.2011.00510.x

61. Jones JA, Lavallee N, Alman J, et al (1993) Caries incidence in patients with dementia. Gerodontology 10:76–82. doi: 10.1111/j.1741-2358.1993.tb00086.x

62. Kim J-M, Stewart R, Prince M, et al (2007) Dental health, nutritional status and recent-onset dementia in a Korean community population. Int J Geriatr Psychiatry 22:850–5. doi: 10.1002/gps.1750

63. Luo J, Wu B, Zhao Q, et al (2015) Association between tooth loss and cognitive function among 3063 Chinese older adults: a community-based study. PLoS One 10:e0120986. doi: 10.1371/journal.pone.0120986

64. Minakuchi S, Takaoka S, Ito J, et al (2006) Factors affecting denture use in some institutionalized elderly people. Spec Care Dentist 26:101–5. doi: 10.1111/j.1754-4505.2006.tb01431.x

65. Miranda LDP, Silveira MF, Oliveira TL, et al (2012) Cognitive impairment, the Mini-Mental State Examination and socio-demographic and dental variables in the elderly in Brazil. Gerodontology 29:e34-40. doi: 10.1111/j.1741-2358.2011.00541.x

66. Mummolo S, Ortu E, Necozione S, et al (2014) Relationship between mastication and cognitive function in elderly in L’Aquila. Int J Clin Exp Med 7:1040–6.

67. Nordenram G, Ryd-Kjellen E, Johansson G, et al (1996) Alzheimer’s disease, oral function and nutritional status. Gerodontology 13:9–16. doi: 10.1111/j.1741-2358.1996.tb00145.x
