# Supplementary material for: Oral hygiene and oral health in older people with dementia: a comprehensive review with focus on oral soft tissues
Source: Clin Oral Investig. 2017 Nov 15;22(1):93–108. doi: 10.1007/s00784-017-2264-2 (PMC5748411; doi:10.1007/s00784-017-2264-2)
Supplement: Supplementary file 2 — (DOCX 81 kb) [file 784_2017_2264_MOESM2_ESM.docx]

**Supplementary Table 1a**: Methodological quality assessment of the included cohort studies with the Newcastle Ottawa Scale

|  | Selection | | | | Comparability | | Outcome | | | Score |
| --- | --- | --- | --- | --- | --- | --- | --- | --- | --- | --- |
| **Cohort study** | Representativeness of cases | Selection of controls | Ascertainment of exposure | Demonstration outcome of interest not present at start of study | Age | Gender | Assessment of oral health | Follow up long enough | Adequacy of follow up | Total |
| Chalmers et al. 2002 [1] | + | - | - | - | + | + | + | + | - | 5 |
| Chalmers et al. 2003 [2] | + | - | - | + | + | + | + | + | + | 7 |
| Chen et al. 2010 [3] | - | + | + | + | - | + | + | + | - | 6 |
| De Souza Rolim et al. 2014b [4] | + | n/a | + | - | - | - | + | - | - | 3 |
| Hatipoglu et al. 2011 [5] | - | - | - | + | - | - | + | + | ? | 3 |
| Hoben et al. 2016 [6] | + | + | - | + | + | ? | - | + | + | 5 |
| Ide et al. 2016 [7] | + | - | + | + | - | - | + | + | + | 6 |
| Ship et al. 1994 [8] | + | + | + | - | + | - | + | + | - | 6 |
| Sumi et al. 2012 [9] | + | n/a | + | - | - | - | + | + | ? | 4 |
| Zenthöfer et al. 2015 [10] | + | + | + | + | + | + | + | + | + | 9 |

NB: + met, - unmet,? unclear, n/a not applicable

**Supplementary Table 1b**: Methodological quality assessment of the included case-control studies with the Newcastle Ottawa Scale

|  | Selection | | | | Comparability | | Exposure | | | Score |
| --- | --- | --- | --- | --- | --- | --- | --- | --- | --- | --- |
| **Case-control study** | Definition of cases | Representativeness of cases | Selection of controls | Definition of controls | Age | Gender | Assessment of oral health | Same method cases & controls | Non response rate | Total |
| Chu et al. 2014 [11] | - | - | - | + | + | + | + | - | + | 5 |
| De Souza Rolim et al. 2014a [12] | + | + | + | + | + | + | + | + | - | 8 |
| Gil-Montoya et al. 2016a [13] | + | + | - | + | - | - | + | + | - | 5 |
| Hoeksema et al. 2016 [14] | + | + | ? | - | - | ? | + | + | - | 4 |
| Kossioni et al. 2012 [15] | + | + | n/a | n/a | + | + | + | + | - | 6 |
| Leal et al. 2010 [16] | - | + | + | + | - | - | + | + | - | 5 |
| Ship et al. 1990 [17] | + | - | + | + | + | - | + | + | - | 6 |
| Warren et al. 1997 [18] | + | + | - | - | + | + | + | + | + | 7 |
| Zenthöfer et al. 2014 [19] | - | - | + | - | + | - | + | + | - | 4 |
| Zenthöfer et al. 2016a [20] | - | + | + | - | - | + | + | + | - | 5 |

NB: + met, - unmet, ? unclear, n/a not applicable

**Supplementary Table 1c**: Methodological quality assessment of the included cross-sectional studies with the Newcastle Ottawa Scale

| Cross-sectional study | Selection | | | | Comparability | | Exposure | | | Score |
| --- | --- | --- | --- | --- | --- | --- | --- | --- | --- | --- |
|  | Definition of cases | Representativeness  of cases | Selection of controls | Definition of controls | Age | Gender | Assessment  of oral health | Same method cases & controls | Nonresponse rate | Total |
| Adam et al. 2006 [21] | - | + | + | - | - | - | + | - | ? | 3 |
| Chapman et al. 1991 [22] | - | + | n/a | n/a | - | - | + | n/a | - | 2 |
| Chen et al. 2013a [23] | - | - | - | - | + | + | - | + | - | 3 |
| Chen et al. 2013b [24] | - | - | - | - | - | + | - | + | - | 2 |
| Chen et al. 2013c [25] | - | - | - | - | - | + | - | + | + | 3 |
| Cohen Mansfield et al. 2002 [26] | - | - | n/a | n/a | - | - | + | n/a | - | 1 |
| Elsig et al. 2013 [27] | + | + | + | - | - | - | + | + | - | 5 |
| Gil-Montoya et al. 2016b [28] | + | + | - | + | + | ? | ? | + | - | 4 |
| Kossioni 2013 [29] | + | + | n/a | n/a | + | + | + | + | - | 6 |
| Lee et al. 2013 [30] | + | - | - | + | + | - | + | + | ? | 5 |
| Philip et al. 2012 [31] | - | - | - | + | - | - | + | + | - | 3 |
| Ribeiro et al. 2012 [32] | + | + | - | + | - | - | + | + | - | 5 |
| Srisilapanan et al. 2013 [33] | - | + | n/a | n/a | - | - | + | n/a | - | 2 |
| Syrjala et al. 2012 [34] | + | + | + | + | + | + | + | + | - | 8 |
| NB: + met, - unmet, ? unclear, n/a not applicable | | | | | | | |  | | |

**Supplementary Table 2**: Methodological quality assessment of the included Controlled Trials with the Delphi list

| **Controlled trial** | Randomization | Concealed treatment allocation | Groups similar at baseline | Eligibility criteria specified | Outcome assessor blinded | Care provider blinded | Patient blinded | Point estimates and measures of variability | Intention to treat analysis |
| --- | --- | --- | --- | --- | --- | --- | --- | --- | --- |
| Fjeld et al. 2014 [35] | yes | yes | yes | yes | yes | ? | ? | yes | no |
| Zenthöfer et al. 2016b [36] | yes | yes | no | yes | yes | no | ? | yes | No |

**Supplementary Table 3**: Assistance need for oral hygiene care of older people with dementia, compared with older people without dementia

| **Study** | **Dementia**  **Number of participants**  **Mean age (SD)** | **No dementia**  **Number of participants**  **Mean age (SD)** | **Assistance need** | **Dementia**  **Prevalence %** | **No dementia**  **Prevalence %** |
| --- | --- | --- | --- | --- | --- |
| Chalmers et al. 2002 | MiD 21  MoD 50  SeD 132 | 21 | Cleaning dentures^[[1]](#endnote-1)^ | MiD: 88.3%  MoD: 97.3% Overall**  SeD: 100.0% | 73.4% |
|  |  |  | Cleaning teeth | MiD: 66.7%  MoD: 57.2% Overall**  SeD: 100.0% | 11.1% |
| Chalmers et al. 2003 | 103  <79y: 82  80+y: 21 | 113  <79y: 88  80+y: 25 | Cleaning dentures  Cleaning teeth | 25.0%**  24.1%** | 0.0%  0.0% |
| Chen et al. 2013a and  Chen et al. 2013c | 46 | 138 | Oral care | 56.5%* | 8.8%* |
| Hoben et al. 2016 | 1606  85.0 (7.5) | 1105  83.4 (10.5) | Daily oral health care by staff | 94.4% | 92.7% |
| Hopcraft et al. 2012 | 105 | 170 | Cleaning teeth; some  Cleaning teeth; total | 35.7%***  21.4% | 14.7%  11.3% |
| Philip et al. 2012 | 84  85.7 (9.6) | 102  84.3 (9.9) | Oral care | 60.3%* | 37.3% |
| Srisilapanan et al. 2013 | 69  75.5 (7.0) | 0 | Oral care | 50.7% | - |

*p≤.05, **p≤.01, ***p≤.001, CI: Cognitive Impairment, MiD: Mild Dementia, MMSE: Mini-Mental State Examination, MoD: Moderate Dementia, SeD: Severe Dementia

**Supplementary Table 4**: The oral mucosal status of older people with dementia, compared with older people without dementia

| **Study** | **Dementia**  **Number of participants**  **Mean age (SD)** | **No dementia**  **Number of participants**  **Mean age (SD)** | **Oral mucosal outcome measure** | **Dementia**  **Prevalence %** | **No dementia**  **Prevalence %** |
| --- | --- | --- | --- | --- | --- |
| Chalmers et al. 2003 | 116 | 116 | Denture stomatitis maxilla | 28.6% | - |
|  |  |  | Angular cheilitis | 5.8% | - |
| Chu et al. 2014 | 59 Mild AD  79.8 (7.4) | 59  79.8 (7.4) | Candidiasis  Lichen planus | 3.6%  1.8% | 3.6%  0.0% |
| De Souza Rolim et al. 2014a&b | 29 Mild AD  75.2 (6.7) | 30  61.2 (11.2) | Candidiasis | 10.3%** | 0.0% |
| Gil-Montoya et al. 2016b | 73 MiD 76.4 (7.5)  66 MoD 77.6 (7.3)  36 SeD 80.4 (6.5) | 156  77.4 (6.9) | Drug-induced xerostomia | MiD 68.5%  MoD 68.5% Overall***  SeD 72.2% | 36.5% |
| Hatipoglu et al. 2011 | 31 AD  67.6 (9.1) | 47  65.3 (7.0) | Stomatitis Maxilla  Stomatitis Mandibula | 59.1%***  18.1%*** | 7.4%  0.0% |
| Kossioni et al. 2012 | 27  76.5 (6.8) | 0 | Stomatitis | 26.0% | - |
| Kossioni et al. 2013 | 23 | 0 | Xerostomia | 34.8% | - |
|  | 76.3 (7.1) |  | Sialorrhea | 0.0% | - |
|  |  |  | Burning mouth | 8.7% | - |
|  |  |  | Dysgeusia | 26.1% | - |
|  |  |  | Oral malodor | 13.0% | - |
| Leal et al. 2010 | 20 MiD with  Medication  69.6 (5.9) | 20 without  medication  68.3 (8.3) | Dry and cracked lips  Fissured tongue  Candidiasis  Ulcerated mucosa  Burning mouth  Dry Mouth | 70.0%*  40.0%*  30.0%  5.0%  30.0%  45.0%* | 30.0%  20.0%  5.0%  5.0%  0.0%  20.0% |
| Warren et al. 1997 | 45 AD  81.6 (6.9) | 133  80.3 (6.8) | Xerostomia | AD 9.1%  OD 22.0%* | 8.4% |
|  | 52 OD  81.4 (7.3) |  | Soft tissue pathology | AD 20.4%  OD 20.4% | 16.8% |

*p≤.05, **p≤.01, ***p≤.001, **AD**: Alzheimer’s disease, **MiD**: Mild Dementia, **OD**: Other dementia’s

**Supplementary Table 5**: Salivary flow in older people with dementia, compared with older people without dementia

| **Study** | **Dementia**  **Number of participants**  **Mean age (SD)** | **No dementia**  **Number of participants**  **Mean age (SD)** | **Salivary flow** | **Dementia**  **Mean (SD)** | **No dementia**  **Mean (SD)** |
| --- | --- | --- | --- | --- | --- |
| Chu et al. 2014 | 59  79.8 (7.4) | 59  79.8 (7.4) | Unstimulated (ml/min) | 0.30 (0.17) | 0.41 (0.28)* |
| Leal et al. 2010 | 20 MiD with medication  69.6 (5.9) | 20 without medication  68.3 (8.3) | Stimulated (ml/min)  Unstimulated (ml/min)  Buffering capacity  pH | 0.69 (0.39)  0.17 (0.17)  0.02 (0.00)  6.71 (0.55) | 0.90 (0.67)  0.73 (0.35)**  0.17 (0.05)***  6.95 (0.42) |
| Ship et al. 1990  2-3y cohort | 28 AD  68.0 (10.0) | 35  70.0 (10.0) | Submandibular Unstimulated (ml/min/gland), mean (SEM)  Submandibular Stimulated (ml/min/gland), mean (SEM)  Parotid Unstimulated (ml/min/gland), mean (SEM)  Parotid Stimulated (ml/min/gland), mean (SEM) | 0.04 (0.00)  0.16 (0.03)  0.06 (0.01)  0.46 (0.06) | 0.09 (0.01)***  0.30 (0.03)***  0.10 (0.02)  0.54 (0.05) |

*p≤.05, **p≤.01, ***p≤.001, **AD**: Alzheimer’s Dementia, **MiD**: Mild Dementia, **OD**: Other Dementia’s

1. With increasing severity of cognitive impairment, residents required significantly more assistance with oral hygiene care and gave caretakers more difficulties with the provision of this care

   1. Chalmers JM, Carter KD, Spencer a J (2002) Caries incidence and increments in community-living older adults with and without dementia. Gerodontology 19:80–94. doi: 10.1111/j.1741-2358.2002.00080.x

   2. Chalmers JM, Carter KD, Spencer AJ (2003) Oral diseases and conditions in community-living older adults with and without dementia. Spec Care Dentist 23:7–17. doi: 10.1111/j.1754-4505.2003.tb00283.x

   3. Chen X, Shuman SK, Hodges JS, et al (2010) Patterns of tooth loss in older adults with and without dementia: a retrospective study based on a Minnesota cohort. J Am Geriatr Soc 58:2300–7. doi: 10.1111/j.1532-5415.2010.03192.x

   4. Rolim T de S, Fabri GMC, Nitrini R, et al (2014) Evaluation of patients with Alzheimer’s disease before and after dental treatment. Arq Neuropsiquiatr 72:919–24. doi: 10.1590/0004-282X20140140

   5. Hatipoglu MG, Kabay SC, Güven G (2011) The clinical evaluation of the oral status in Alzheimer-type dementia patients. Gerodontology 28:302–6. doi: 10.1111/j.1741-2358.2010.00401.x

   6. Hoben M, Poss JW, Norton PG, Estabrooks CA (2016) Oral/dental items in the resident assessment instrument – minimum Data Set 2.0 lack validity: results of a retrospective, longitudinal validation study. Popul Health Metr 14:36. doi: 10.1186/s12963-016-0108-y

   7. Ide M, Harris M, Stevens A, et al (2016) Periodontitis and Cognitive Decline in Alzheimer’s Disease. PLoS One 11:e0151081. doi: 10.1371/journal.pone.0151081

   8. Ship JA, Puckett SA (1994) Longitudinal study on oral health in subjects with Alzheimer’s disease. J Am Geriatr Soc 42:57–63.

   9. Sumi Y, Ozawa N, Michiwaki Y, et al (2012) [Oral conditions and oral management approaches in mild dementia patients]. Nihon Ronen Igakkai Zasshi 49:90–8.

   10. Zenthöfer A, Cabrera T, Rammelsberg P, Hassel AJ (2016) Improving oral health of institutionalized older people with diagnosed dementia. Aging Ment Health 20:303–8. doi: 10.1080/13607863.2015.1008986

   11. Chu CH, Ng A, Chau AMH, Lo ECM (2015) Oral health status of elderly chinese with dementia in Hong Kong. Oral Health Prev Dent 13:51–7. doi: 10.3290/j.ohpd.a32343

   12. de Souza Rolim T, Fabri GMC, Nitrini R, et al (2014) Oral infections and orofacial pain in Alzheimer’s disease: a case-control study. J Alzheimers Dis 38:823–9. doi: 10.3233/JAD-131283

   13. Gil-Montoya JA, Sánchez-Lara I, Carnero-Pardo C, et al (2017) Oral Hygiene in the Elderly with Different Degrees of Cognitive Impairment and Dementia. J Am Geriatr Soc 65:642–647. doi: 10.1111/jgs.14697

   14. Hoeksema AR, Peters LL, Raghoebar GM, et al (2016) Oral health status and need for oral care of care-dependent indwelling elderly: from admission to death. Clin Oral Investig. doi: 10.1007/s00784-016-2011-0

   15. Kossioni AE, Kossionis GE, Polychronopoulou A (2012) Oral health status of elderly hospitalised psychiatric patients. Gerodontology 29:272–83. doi: 10.1111/j.1741-2358.2012.00633.x

   16. Leal SC, Bittar J, Portugal A, et al (2010) Medication in elderly people: its influence on salivary pattern, signs and symptoms of dry mouth. Gerodontology 27:129–33. doi: 10.1111/j.1741-2358.2009.00293.x

   17. Ship JA, DeCarli C, Friedland RP, Baum BJ (1990) Diminished submandibular salivary flow in dementia of the Alzheimer type. J Gerontol 45:M61-6.

   18. Warren JJ, Chalmers JM, Levy SM, et al (1997) Oral health of persons with and without dementia attending a geriatric clinic. Spec Care Dentist 17:47–53. doi: 10.1111/j.1754-4505.1997.tb00866.x

   19. Zenthöfer A, Schröder J, Cabrera T, et al (2014) Comparison of oral health among older people with and without dementia. Community Dent Health 31:27–31.

   20. Zenthöfer A, Baumgart D, Cabrera T, et al (2017) Poor dental hygiene and periodontal health in nursing home residents with dementia: an observational study. Odontology 105:208–213. doi: 10.1007/s10266-016-0246-5

   21. Adam H, Preston AJ (2006) The oral health of individuals with dementia in nursing homes. Gerodontology 23:99–105. doi: 10.1111/j.1741-2358.2006.00118.x

   22. Chapman PJ, Shaw RM (1991) Normative dental treatment needs of Alzheimer patients. Aust Dent J 36:141–4. doi: 10.1111/j.1834-7819.1991.tb01343.x

   23. Chen X, Clark JJJ, Naorungroj S (2013) Oral health in older adults with dementia living in different environments: a propensity analysis. Spec Care Dentist 33:239–47. doi: 10.1111/scd.12006

   24. Chen X, Clark JJJ, Naorungroj S (2013) Oral health in nursing home residents with different cognitive statuses. Gerodontology 30:49–60. doi: 10.1111/j.1741-2358.2012.00644.x

   25. Chen X, Clark JJ, Chen H, Naorungroj S (2015) Cognitive impairment, oral self-care function and dental caries severity in community-dwelling older adults. Gerodontology 32:53–61. doi: 10.1111/ger.12061

   26. Cohen-Mansfield J, Lipson S (2002) The underdetection of pain of dental etiology in persons with dementia. Am J Alzheimers Dis Other Demen 17:249–53. doi: 10.1177/153331750201700404

   27. Elsig F, Schimmel M, Duvernay E, et al (2015) Tooth loss, chewing efficiency and cognitive impairment in geriatric patients. Gerodontology 32:149–56. doi: 10.1111/ger.12079

   28. Gil-Montoya JA, Barrios R, Sánchez-Lara I, et al (2016) Prevalence of Drug-Induced Xerostomia in Older Adults with Cognitive Impairment or Dementia: An Observational Study. Drugs Aging 33:611–8. doi: 10.1007/s40266-016-0386-x

   29. Kossioni AE, Kossionis GE, Polychronopoulou A (2013) Self-reported oral complaints in older mentally ill patients. Geriatr Gerontol Int 13:358–64. doi: 10.1111/j.1447-0594.2012.00907.x

   30. Lee KH, Wu B, Plassman BL (2013) Cognitive function and oral health-related quality of life in older adults. J Am Geriatr Soc 61:1602–7. doi: 10.1111/jgs.12402

   31. Philip P, Rogers C, Kruger E, Tennant M (2012) Oral hygiene care status of elderly with dementia and in residential aged care facilities. Gerodontology 29:e306-11. doi: 10.1111/j.1741-2358.2011.00472.x

   32. Ribeiro GR, Costa JLR, Ambrosano GMB, Garcia RCMR (2012) Oral health of the elderly with Alzheimer’s disease. Oral Surg Oral Med Oral Pathol Oral Radiol 114:338–43. doi: 10.1016/j.oooo.2012.03.028

   33. Srisilapanan P, Jai-Ua C (2013) Oral health status of dementia patients in Chiang Mai Neurological Hospital. J Med Assoc Thai 96:351–7.

   34. Syrjälä A-MH, Ylöstalo P, Ruoppi P, et al (2012) Dementia and oral health among subjects aged 75 years or older. Gerodontology 29:36–42. doi: 10.1111/j.1741-2358.2010.00396.x

   35. Fjeld KG, Mowe M, Eide H, Willumsen T (2014) Effect of electric toothbrush on residents’ oral hygiene: a randomized clinical trial in nursing homes. Eur J Oral Sci 122:142–8. doi: 10.1111/eos.12113

   36. Zenthöfer A, Meyer-Kühling I, Hufeland A, et al (2016) Carers’ education improves oral health of older people suffering from dementia - results of an intervention study. Clin Interv Aging 11:1755–1762. doi: 10.2147/CIA.S118330 [↑](#endnote-ref-1)
